# Experimental study and modeling of atomic-scale friction in zigzag and armchair lattice orientations of MoS_2_

**DOI:** 10.1080/14686996.2016.1165584

**Published:** 2016-04-25

**Authors:** Meng Li, Jialin Shi, Lianqing Liu, Peng Yu, Ning Xi, Yuechao Wang

**Affiliations:** ^a^ State Key Laboratory of Robotics, Shenyang Institute of Automation, Chinese Academy of Sciences, Shenyang110016, PRChina; ^b^ University of the Chinese Academy of Sciences, Beijing100049, PRChina; ^c^ Department of Electrical and Computer Engineering, Michigan State University, East Lansing, Michigan48824, PRUSA

**Keywords:** MoS_2_, atomic-scale friction, two-dimensional model, lattice orientation, 40 Optical, magnetic and electronic device materials, 105 Low-Dimension (1D/2D) materials, 100 Materials, 212 Surface and interfaces, 200 Applications, 303 Mechanical / Physical processing, 300 Processing / Synthesis and Recycling

## Abstract

Physical properties of two-dimensional materials, such as graphene, black phosphorus, molybdenum disulfide (MoS_2_) and tungsten disulfide, exhibit significant dependence on their lattice orientations, especially for zigzag and armchair lattice orientations. Understanding of the atomic probe motion on surfaces with different orientations helps in the study of anisotropic materials. Unfortunately, there is no comprehensive model that can describe the probe motion mechanism. In this paper, we report a tribological study of MoS_2_ in zigzag and armchair orientations. We observed a characteristic power spectrum and friction force values. To explain our results, we developed a modified, two-dimensional, stick-slip Tomlinson model that allows simulation of the probe motion on MoS_2_ surfaces by combining the motion in the Mo layer and S layer. Our model fits well with the experimental data and provides a theoretical basis for tribological studies of two-dimensional materials.

## Graphical abstract

**Figure:**
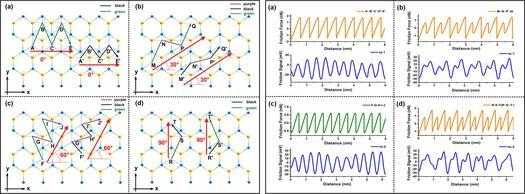

## 1. Introduction

Two-dimensional materials with strong in-plane covalent bonding and weak van der Waals interaction are the most common class of crystalline structures that can be easily exfoliated into atomically thin layers. Achievements in fabrication have led to a new research field devoted to the study of their novel physical, chemical and mechanical properties,[1–6] which have fascinated the scientific community. Among the family of two-dimensional materials, the form that attracts the most interest is graphene, which has shown superior carrier mobility of up to 200,000 cm^2^V^–1^ and record thermal conductivity and stiffness.[7–9] The discovery of graphene has also unveiled other layered-structure materials, which include the graphene analogs hexagonal boron nitride (h-BN), black phosphorus, and transition metal dichalcogenides (TMDC). For instance, the perfectly flat and electrically insulating properties allow the use of h-BN as an alternative substrate and excellent gate dielectric.[10,11] A predicted direct bandgap of 2 eV and its anisotropic nature make black phosphorus an ideal material for the infrared regime and new types of plasmonic devices.[12,13]

Among the two-dimensional materials, graphene, with inherent zero-band gap,[14] usually requires complex bandgap engineering processes to fabricate field-effect transistors (FET).[15,16] The wide bandgap of h-BN makes it unsuitable for electronic applications.[17] Black phosphorus, however, is chemically inert in atmosphere, and its monolayer form is expected to be destroyed upon exposure to air,[18] which remains an important challenge restricting its future applications. By comparison, several TMDCs have sizable bandgaps of 1–2 eV and possess novel properties that are complementary to, yet distinctive from, those of graphene, ranging from electronic, optical, mechanical, chemical and thermal properties.[19,20] Furthermore, TMDCs are generally air-stable and naturally abundant,[21] rendering them promising channel materials to replace silicon.

In particular, MoS_2_, a TMDC, is useful in device applications due to its satisfactory bandgaps, good flexibility, thermal stability, excellent carrier mobility, and compatibility with silicon CMOS (Complimentary Metal Oxide Semiconductor),[22,23] and is being heavily investigated for wide-ranging application in electronics and optoelectronics.[24–27] For example, the high on-off current ratio, up to 200 cm^2^ V^–1^ carrier mobility and good sub-threshold swing have been utilized to fabricate more complex, two-dimensional, hetero-CMOS inverters with low power consumption.[28] An indirect-to-direct bandgap transition occurs when bulk MoS_2_ is thinned to a monolayer, which induces strong photoluminescence (PL) with sensitive photoresponse, thus rendering it attractive for optoelectronic devices.[29,30] In sum, the intriguing prospect of the potential nanoelectronic applications, which may take advantage of the novel physical properties, has inspired researchers to explore the unknown characteristics of MoS_2_.

Recently, intensive theoretical research on MoS_2_ nanoribbons has shown that armchair nanoribbons are nonmagnetic and semiconducting,[31] whereas zigzag nanoribbons are ferromagnetic metallic.[32] Thus, most of the aforementioned electrical and optical applications largely depend on the edge state because metallic edges may result in current leakage and thereby immensely hinder normal device function.[33] However, for piezotronics, zigzag edge is preferable because it induces and accumulates piezoelectric polarization charges to produce piezoelectric current and voltage responses due to its ferromagnetic property.[34] In view of the fact that different chirality directly leads to distinct properties and practical applications, it is essential to extensively investigate these two lattice orientations, towards finding a convenient method to effectively differentiate them in the future.

The specific chirality of a material can be determined by the lattice orientation detection technique,[35,36] which presents the atomic resolution of the corresponding material and enables us to investigate the unknown microscopic domains. Current lattice orientation detection techniques include transmission electron microscopy (TEM), scanning tunneling microscopy (STM) and atomic force microscopy (AFM).[37–40] TEM and STM techniques require either extremely expensive equipment, ultra-high vacuum (UHV) environment or additional gold coating procedures to produce a conductive substrate, which greatly increases the difficulty of detecting multiple lattice orientation. AFM is also able to achieve a high-resolution atomic image, and has the advantage over TEM and STM that it can simultaneously acquire the lattice orientation and friction information through the atomic image of the material surface in lateral force microscopy (LFM) mode. Meanwhile, as a lubricant material, the tribological properties of MoS_2_ have a significant impact on many fields of science and technology, and the relevant research mainly focuses on the friction force of interlayer sliding [41–43] or the friction motion on various substrates.[44] Atomic-scale friction has been studied but knowledge is not sufficient. Research on atomic-scale friction between the probe and surface is scarce and does not address the relationship between friction and lattice orientation. In view of the shortcomings in TEM and STM techniques and the enormous significance of atomic-scale tribological research, AFM is more suitable for our study and is employed to obtain the friction information.

This study aims to carry out research on atomic-scale friction of MoS_2_ and to work towards identifying lattice orientation, and is the first study of the friction variation and friction force in zigzag and armchair lattice orientations of MoS_2_ using AFM and LFM. A modified model is established to explain thoroughly the motion mechanism of the probe on the MoS_2_ surface in zigzag and armchair orientations. For a better comparison of the friction signal variation, fast Fourier transform (FFT) is performed. Finally, measurements of the friction force in the experiment and simulation are conducted to explore uncharted territories in the atomic-scale friction of MoS_2_.

## 2. Modeling of the lateral friction in zigzag and armchair orientations

In the lateral friction experiment, stick-slip movement occurs between the probe and MoS_2_ surface.[45] To derive the relationship between the friction force and the lattice orientation and to further establish a comprehensive model of probe motion, a two-dimension Tomlinson model,[46] which describes the movement in idealized LFM constant-force mode, is adopted for the simulation modeling:

(1) $$\begin{array}{c} {\text{m}}_{\text{x}}{\ddot{x}}_{\text{t}}{\;\text{= k}}_{\text{x}}\left(x_{M}-x_{t}\right)-\frac{\partial V(x_{t},y_{t})}{\partial x_{t}}-\gamma_{x}{\dot{x}}_{t},\\ {\text{m}}_{\text{y}}{\ddot{y}}_{\text{t}}{\;\text{= k}}_{\text{y}}\left(y_{M}-y_{t}\right)-\frac{\partial V(x_{t},y_{t})}{\partial y_{t}}-\gamma_{y}{\dot{y}}_{t}, \end{array}$$

where m_x_ and m_y_ are the effective mass of the system, *x*_*t*_ and *y*_*t*_ are the actual positions of probe at particular time, *x*_*M*_ and *y*_*M*_ are the equilibrium positions of the probe, k_x_ and k_y_ are the elasticity of the probe, and *γ*_*x*_ and *γ*_*y*_ are the damping constants of the system. In theory, the degree of freedom for the motion of atoms is high in two-dimensional systems in which non-adiabatic motion is avoided.[47] In our two-dimensional Tomlinson model, the sweeping of the probe can be regarded as adiabatic.

**Figure 1.**
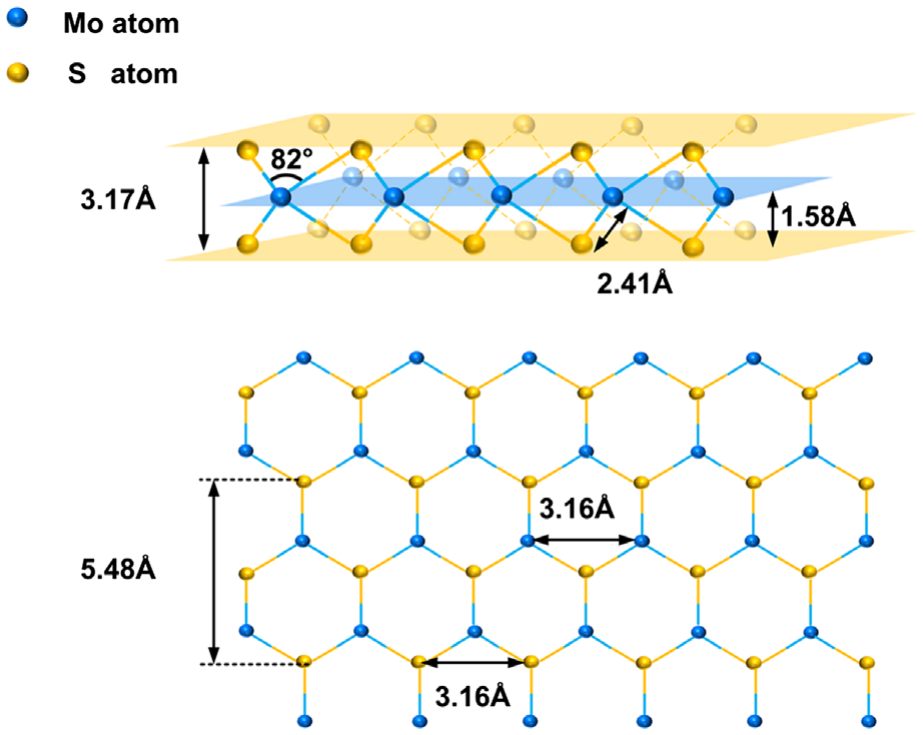
Lattice constants of MoS_2_. The top part shows a lateral view of MoS_2_, while the bottom part depicts a top view. The yellow and blue spheres indicate S and Mo atoms of MoS_2_, respectively.

To obtain the solutions of Equation (1), an appropriate and simple interaction potential *V*(*x*_*t*_, *y*_*t*_) has to be selected. The original interaction potential is described as:

(2) $${\text{V(x}}_{t},y_{t})=V_{0}\cos\left(\frac{2\pi }{a_{x}}x_{t}\right)\cos\left(\frac{2\pi }{a_{y}}y_{t}\right)$$

where *a*_*x*_ and *a*_*y*_ are the lattice constants of S layer in a MoS_2_ film. Typical LFM parameters are:[46] *V*_0_ = 1.0*ev*, *k*_*x*_ = *k*_*y*_ = 10Nm^−1^,*V*_*m*_=400 Å S^−1^, *V*_0_ is the initial value of tip-sample interaction, and *V*_*m*_ is the scan speed. Then, the damping constant of the system can be obtained $\gamma_{x}=\gamma_{y}=2\sqrt{k_{x}m_{x}}\approx 10^{-3}$Ns/m. MoS_2_ film can be envisioned as a sandwich-like slab that consists of two individual atomic layers, a Mo layer and an S layer. As shown in the inset of Figure 1, Mo and S atoms are covalently linked in a sandwich structure. According to the literature, the characteristic value of MoS_2_ can be approximated as follows: in the same layer, the distances between Mo atom and S atom and between the two S atoms are 3.16 Å and 2.41 Å, respectively; however, in different layers, the perpendicular distance between two S atoms and between S and Mo atoms is 3.17 Å and 1.58 Å, respectively. The bond angle between two S atoms and a Mo atom is 82°.

Given the stick-slip movement, the probe shuttles between the upper layer (S layer) and lower layer (Mo layer) in the actual LFM experiment. Although the interaction between the probe and lower layer might be weaker compared to that of the upper layer, it should not be ignored to establish a comprehensive motion model. In the following two sections, the probe motion in zigzag and armchair orientations combining two layers will be discussed in detail.

### 2.1. Motion in zigzag orientation

**Figure 2.**
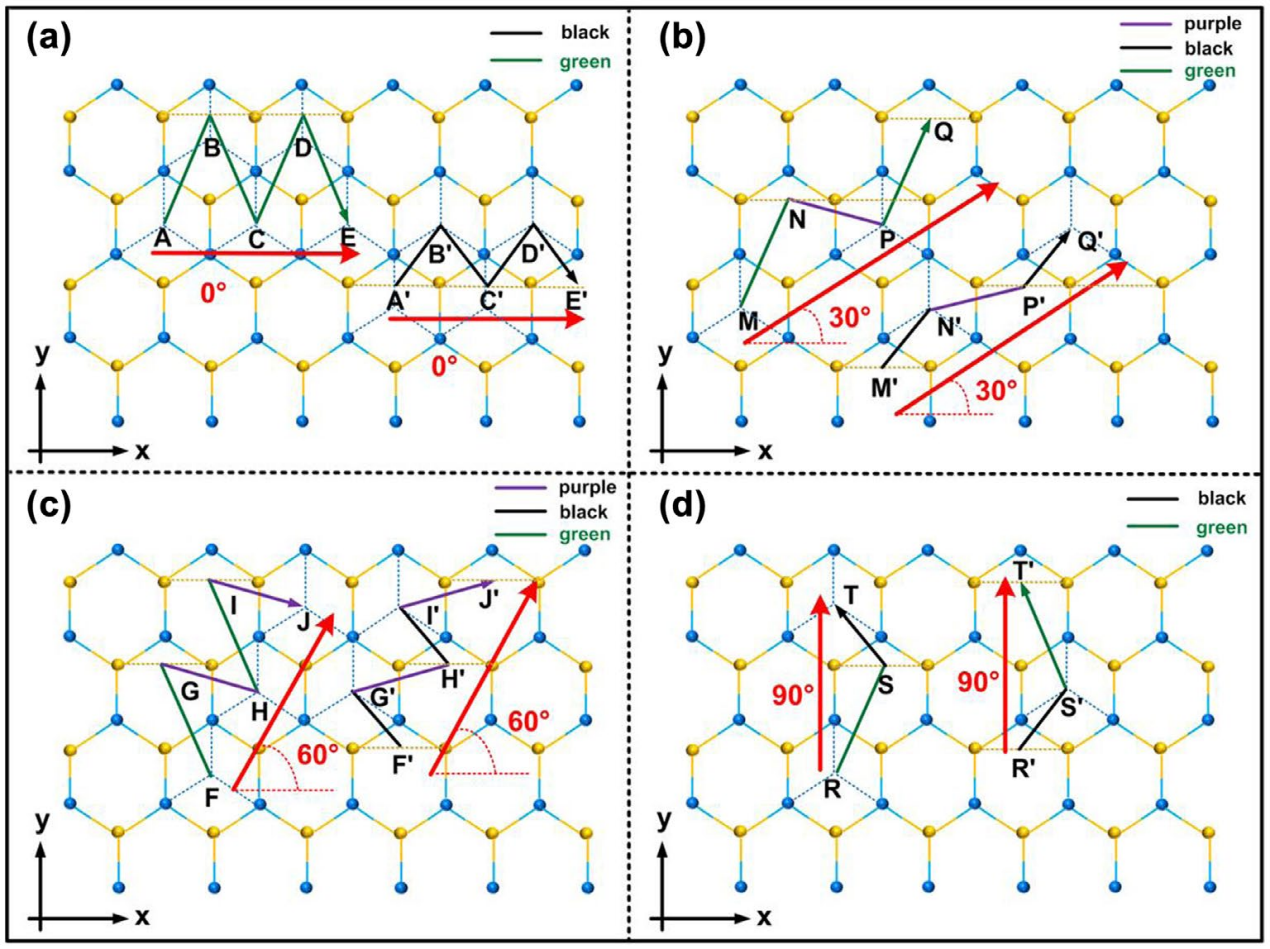
Moving trajectories in various lattice orientations: (a) zz-1 orientation; (b) ac-1 orientation; (c) zz-2 orientation; (d) ac-2 orientation. The black, green and purple arrow lines indicate the moving trajectories of the probe along different lattice orientations. The red arrow lines indicate the scan direction of the probe. The dashed yellow lines connect two adjacent S atoms via the central points of S crystal cell. The dashed blue lines connect two adjacent Mo atoms via the central points of Mo crystal cell.

For zigzag orientation, the probe moves along two distinct paths due to the disparity in the relative position of the MoS_2_ lattice structure, which are the first type zigzag (zz-1) and the second type zigzag (zz-2), as depicted in Figures 2(a) and (c), respectively. The scan direction of zz-1 is defined as 0°. For zz-2, the scan direction is 60°. In view of the characteristic of stick-slip movement, we assume the probe moves via the central points in each layer. Then, considering the two different types of atoms in MoS_2_, the probe motion in each path of zigzag orientation can be further divided into two distinct cases: one case starts from the central points of the Mo crystal cell and terminates at the central points of the S crystal cell; the other case is the opposite. For instance, in the case of zz-1, as shown in Figure 2(a), two moving trajectories are possible, A-B-C-D-E or A′-B′-C′-D′-E′. For A-B-C-D-E, the trajectory consists of various lines connecting the central points of the Mo crystal cell and that of the adjacent S crystal cell. In contrast, the component lines of A′-B′-C′-D′-E′ connect the central points of the S crystal cell to that of the adjacent Mo crystal cell. The moving trajectories of zz-2, F-G-H-I-J, and F′-G′-H′-I′-J′, displayed in Figure 2(c), are constructed in the same way. In both paths, these lines are marked with different colors (black, green and purple) according to their specific lengths. The calculation is discussed in the third part of this chapter.

### 2.2. Motion in armchair orientation

In a similar way, the moving path of armchair orientation can be divided into two distinct paths: the first type armchair (ac-1) and the second type armchair (ac-2), as depicted in Figures 2(b) and (d). The scan directions of these two paths are 30° and 90°, respectively. Each path contains two different trajectories. As shown in Figures 2(b) and (d), in armchair orientation, the probe moves along M-N-P-Q or M′-N′-P′-Q′ and R-S-T or R′-S′-T′. The component of each trajectory is the same as in zigzag orientation discussed above: the line segments between the central points of S or Mo crystal cell and that of their adjacent crystal cell. These lines segments are also marked in different colors in terms of length. Notably, as shown in Ffigure 2(d), the length of the moving trajectories R-S-T and R′-S′-T′ in the second type of armchair orientation are equal due to the specific lattice symmetry.

### 2.3. Simulation results

According to the analysis above, the movements of the probe in both S layer and Mo layer must be considered together. The lattice constants in the original interaction potential described in Equation (2) need to be modified because they simply reflect the movement in S layers. To obtain the actual lattice constants combining the movement in two layers, the accurate length of the various trajectories should be calculated first. Based on the triangle cosine theorem, the length of the line segments in different colors can be obtained by taking these parameters into the calculation. Furthermore, the length of various trajectories, R, both in zigzag and armchair orientations, can be obtained by simple addition. The results are summarized in Table 1.

**Table 1.** Results of the trajectory length in various lattice orientations.

Color	Computation result (Å)	Lattice orientation	Expression of motion period	R (Å)
Black (b)	2.85	zz-1	2 x b / 2 x g	5.7 / 8.12
Green (g)	4.06	zz-2	b + p / g + p	6.51 / 7.72
Purple (p)	3.66	ac-1	b + p / g + p	6.51 / 7.72
		ac-2	b + g	6.91

Based on the data, the original interaction potential can be modified as:

(3) $${\text{V(x}}_{t},y_{t})=V_{0}\cos\left(\frac{2\pi }{R^{*}}x_{t}\right)\cos\left(\frac{2\pi }{\frac{R^{*}}{a_{x}}a_{y}}y_{t}\right)$$

Where *R* ^*^ is the actual lattice constant considering the moving trajectory R in various lattice orientations. The expression of *R* ^*^ is as follows:

(4) $${\text{R}}^{*}\;\text{= Rcos}\theta$$

where *θ* is the corresponding lattice orientation. For zz-1, zz-2, and ac-1, the values of *θ* are 0°, 60°, and 30°, respectively. For ac-2, considering the particularity of the cosine function (cos90° = 0), 90° is treated as 30°.

**Figure 3.**
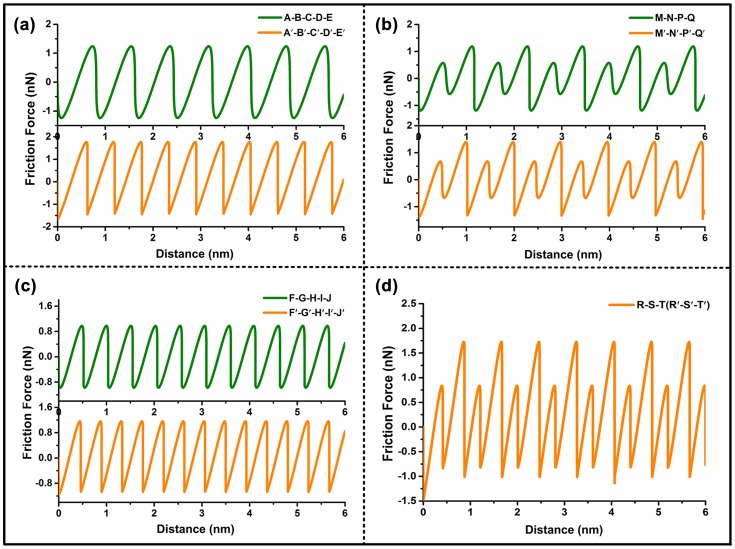
Simulation results of the lateral friction for various lattice orientations of MoS_2_: (a) zz-1 orientation; (b) ac-1 orientation; (c) zz-2 orientation; (d) ac-2 orientation. The simulated waves marked in green indicate the first-case trajectory in each path of zigzag orientation or armchair orientation. The simulated waves marked in orange indicate the second-case trajectory in each path of zigzag orientation or armchair orientation.

Then, simulations of the lateral force friction under zigzag and armchair orientations are performed using Matlab software (MathWorks, USA, version 7.0). The simulation results are displayed in Figure 3.

## 3. Experimental method

The substrate material used for room-temperature tests was silicon covered with a 285-nm-thick oxide. To improve the measurement accuracy, two types of multilayer MoS_2_ film samples were fabricated. The first type, labeled sample-1, was grown by chemical vapor deposition at the Shanghai Institute of Optics and Fine Mechanics. The original solution was diluted three times and then sonicated for 12 minutes at 59 Hz in an ultrasonic oscillator (SK5210 LHC). One 2 µl drop was then cast on a freshly cleaned silicon substrate and dried at room temperature. The second film type, labeled sample-2, was mechanically peeled from bulk MoS_2_ (provided by XFNANO, Nanjing, China).

**Figure 4.**
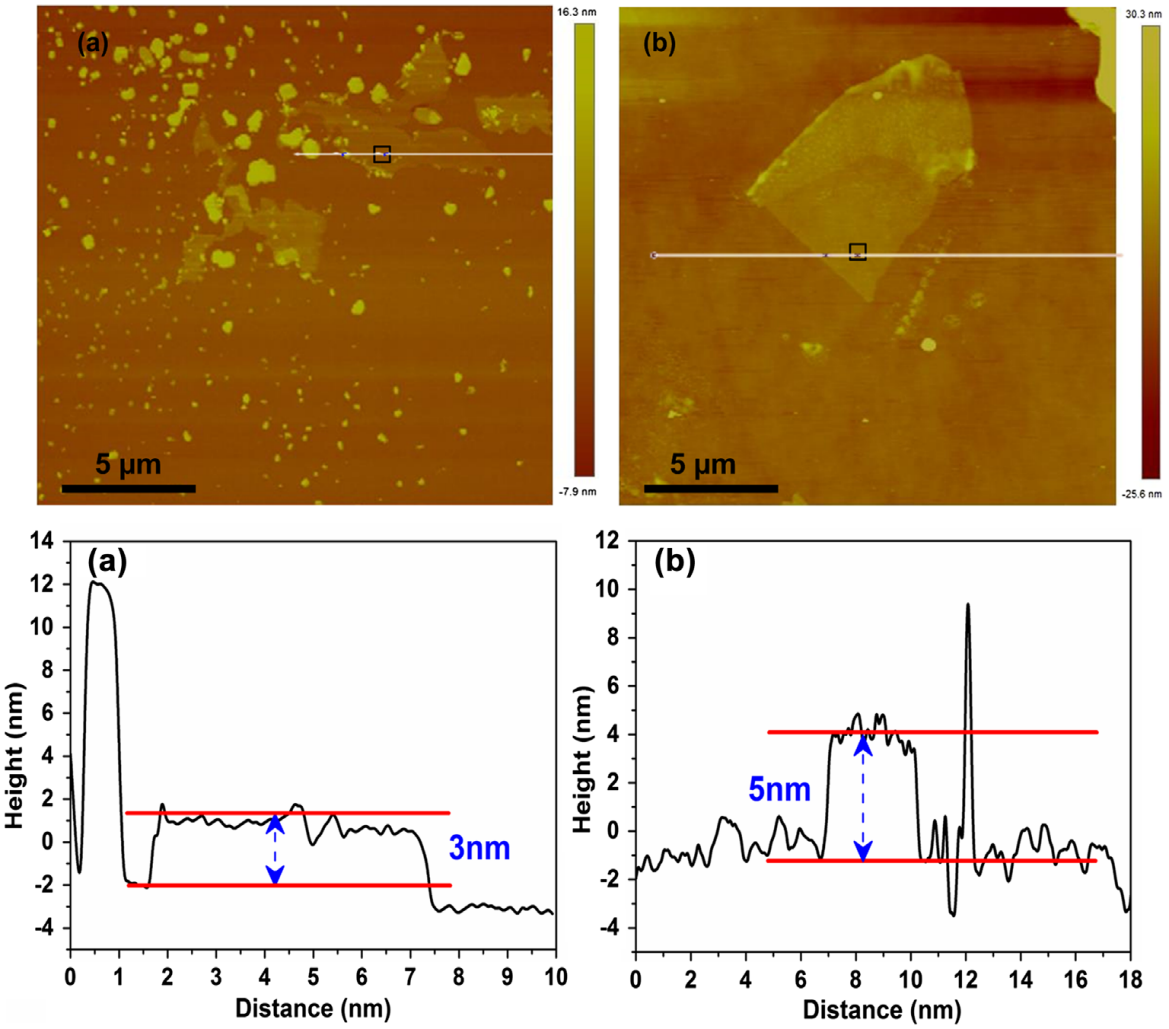
(a, b) Surface morphology of the multilayer MoS_2_ films: (a) sample-1; (b) sample-2. (c, d) Height profile along the white line in (c) panel a; (d) panel b. The black rectangles indicate the scan areas.

AFM was utilized to observe and analyze the surface morphology. A flat and clean surface of the sample is required to satisfy the adiabatic condition and to ensure the experiment proceeds smoothly. For this purpose, we prepared flat and smooth pieces of film before the experiment. For the first type of MoS_2_ film, shown in Figure 4(a), we use a relatively flatter and larger sample, whose size is approximately 7.5 × 5.3 µm and average height is approximately 3 nm, indicating that it is a five-layer MoS_2_. For the second type of MoS_2_ film, shown in Figure 4(b), the size is approximately 9 × 6 µm and the average height is approximately 5 nm, suggesting it is an eight-layer MoS_2_.

**Figure 5.**
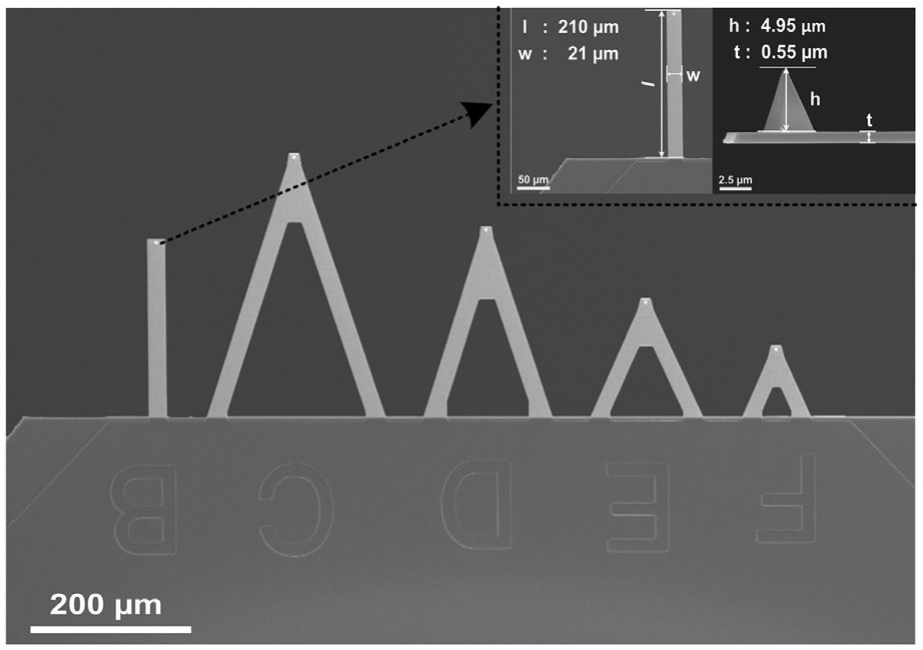
Scanning electron microscopy image of the probe used for the LFM experiment. *l*, *w*, *h*, and *t* define the length, width, height and thickness of the probe, respectively.

Subsequently, to acquire the atomic image, an LFM experiment was performed in the contact mode of a multimode AFM in air under ambient conditions (25–60% relative humidity, 20–50 °C). To guarantee a rigorous contrast between the two samples, LFM experiments for two cases were performed under the same conditions. We adopted the same type of probe, i.e. a normal contact silicon nitride AFM probe (MLC-B, Bruker, Inc.[48]) The length, width, height, radius, and thickness of the probe were 210 µm, 21 µm, 4.95 µm, 10 nm and 0.5 m, respectively, as shown in the inset of Figure 5. The spring constant of the probe was 0.02 N m^–1^. During the experiment for two cases, the scan size and rate were 10 nm and 29.5 Hz, respectively. The LFM experiment typically achieved the best atomic-scale imaging under 90° scanning. The scan angle in two cases remained constant at 90°. In addition, the proportional gain, integral gain and setpoint value were kept at 0.2, 0.3 and 0.5, respectively. Finally, the total number of the lines for each atomic image, i.e. the pixel resolution of the image, was 256.

## 4. Results and discussion

### 4.1. Atomic images of two types of MoS_2_ multilayers

**Figure 6.**
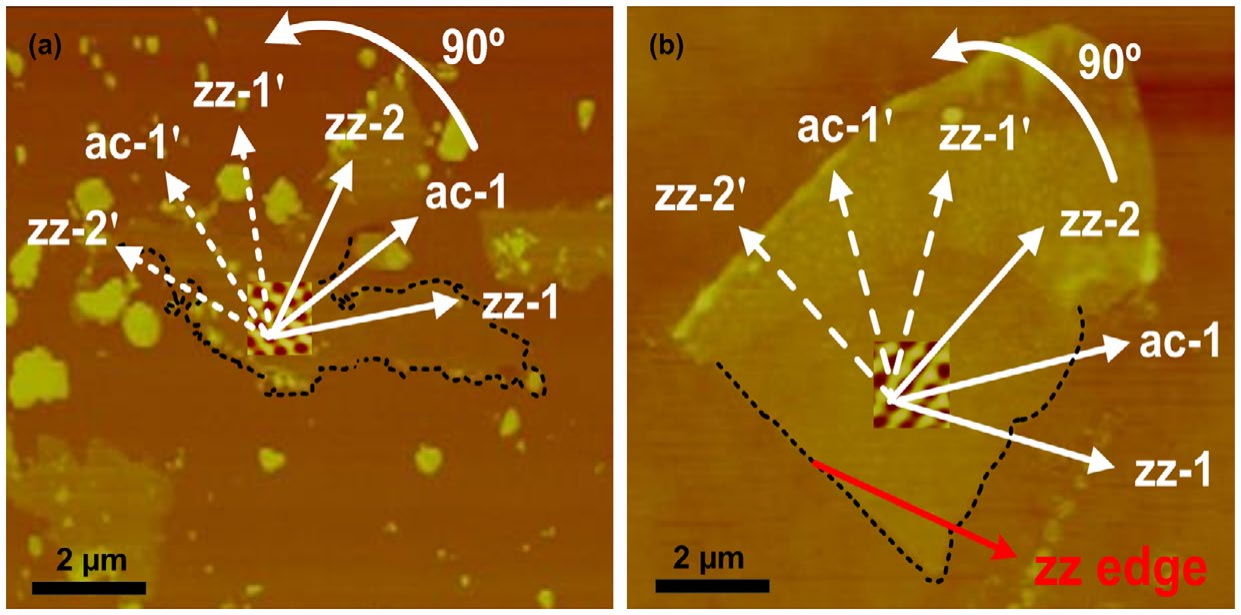
Lattice orientation identification of the multilayer MoS_2_ films: (a) sample-1; (b) sample-2. The white arrow lines indicate the zigzag and armchair orientations in the atomic image. The dotted white arrow lines indicate the actual zigzag and armchair orientations of MoS_2_ films after rotating 90°. The black dotted lines depict the partial edges of MoS_2_ films.

After FFT filtering, atomic images for the two types of MoS_2_ films were acquired. The surface morphology and atomic images of the central parts in MoS_2_ films are displayed in Figure 6(a) and (b). Because the scanning angle in two cases was constant at 90°, the lattice structure was achieved by rotating the original lattice by 90° either clockwise or counter-clockwise (the obtained results were the same in both cases). In addition, for a single crystal, the lattice distribution of the entire sample is continuous and unbroken. Therefore, the edge state is consistent with the inner structure. The right sharp edge of the mechanical exfoliated single crystal MoS_2_ film can be safely deduced through the central atomic distribution image because it is parallel to zz-2′ orientation, as indicated by the red arrow in Figure 6(b). After acquiring these atomic images, the useful information they contain is further extracted and discussed in detail in the following section.

### 4.2. Characteristics of actual lateral friction signals

**Figure 7.**
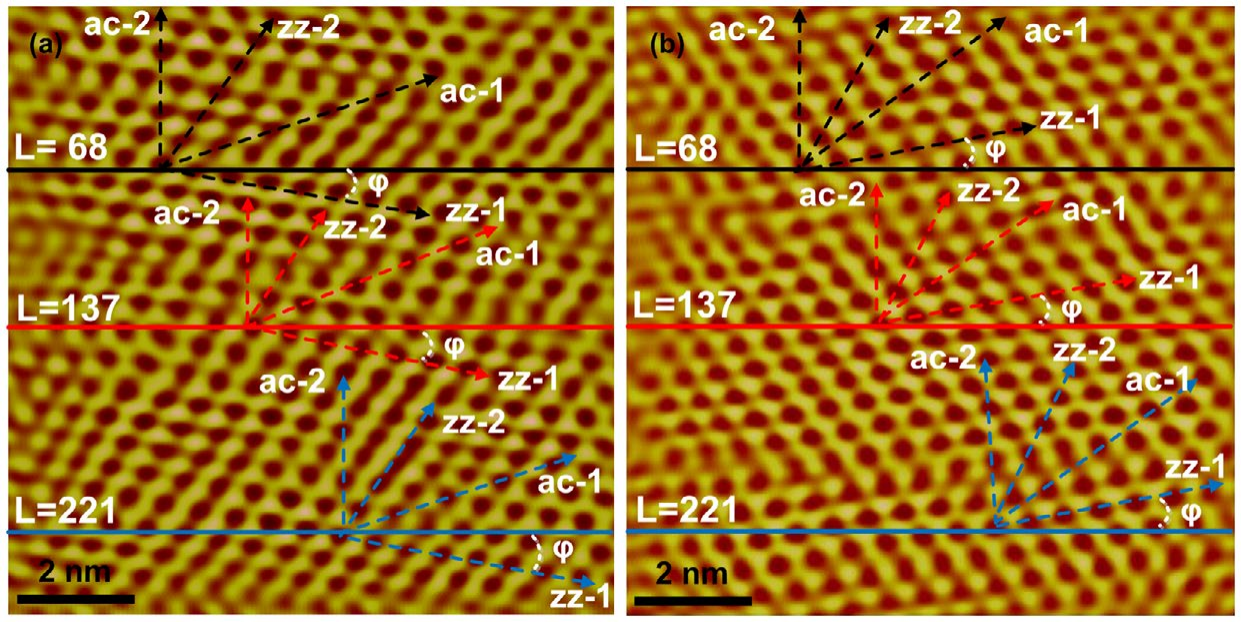
Atomic images of multilayer MoS_2_ films. (a) Sample-1 and (b) Sample-2. The black, red and blue lines indicate the 68th, 137th and 221st rows of the atomic image, respectively. The four dashed lines marked different colors indicate zz-1, zz-2, ac-1 and ac-2 orientations at different locations, respectively.

After discerning the different lattice orientations, the lateral friction signal waves of the corresponding film can be obtained by pulling a line along each angle in the atomic image, as displayed in Figures 7(a) and (b).

**Figure 8.**
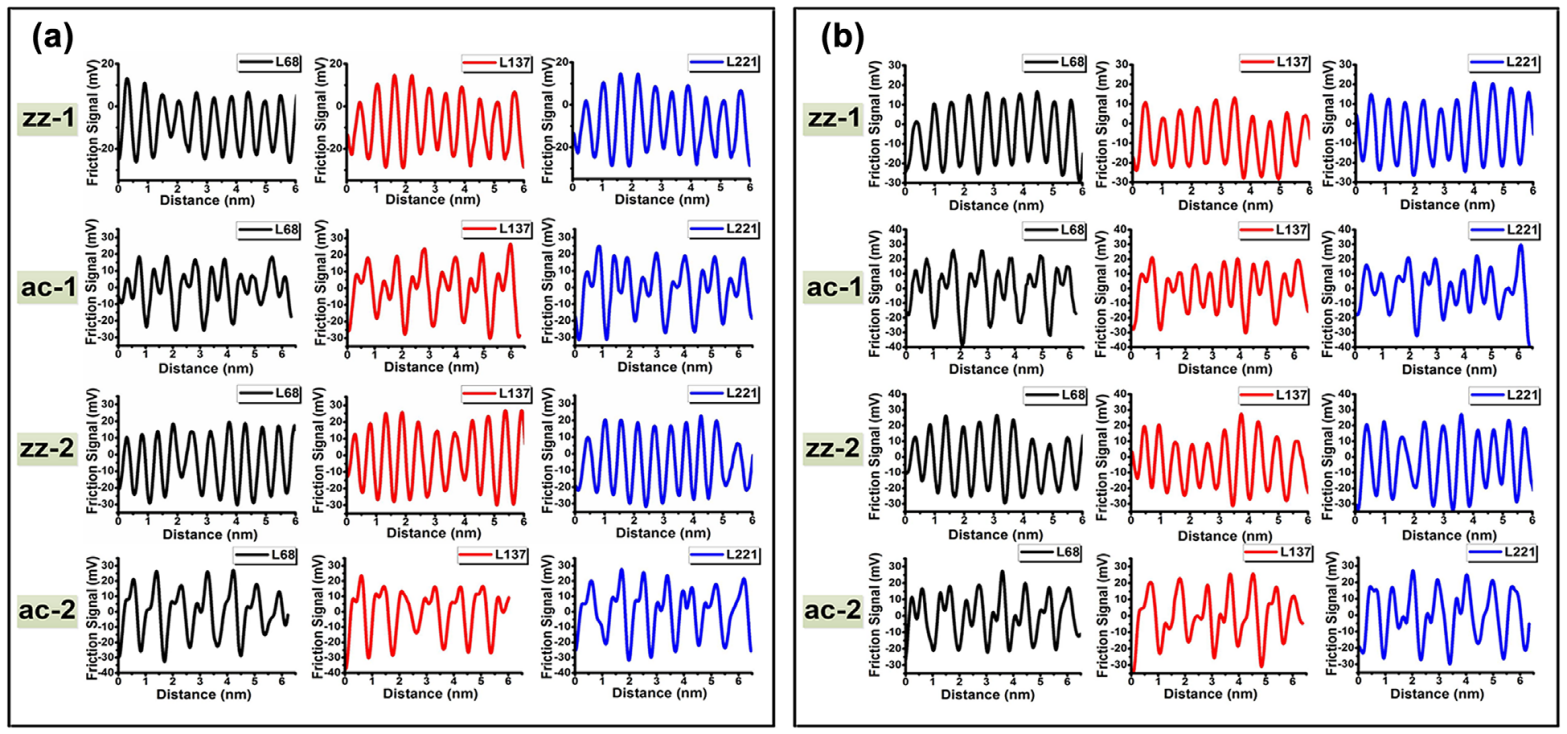
Lateral friction signal of multilayer MoS_2_ films. (a) Lateral friction signal of sample-1. (b) Lateral friction signal of sample-2. The waves in black, red and blue indicate the friction signal obtained from 68th, 137th and 221st rows of the atomic image, respectively.

For the sake of accuracy, each angle was processed at three different locations of the friction atomic image. Because the total number of the rows per image is 256, the three locations can be differentiated by the number of rows they locate. Three distinct locations are colored in black, red and blue. In addition, φ, representing the actual location of the films, is the angle between the 0° scanning direction and 0° lattice orientation, which remains constant in the same friction atomic image. The values of φ are approximately 10° and 7°. By pulling the line along different angles of 0° and 60° or 30° and 90°, lateral friction signal waves of zigzag and armchair lattice orientations can be obtained, respectively. The lateral friction signal waves in two cases are plotted in Figure 8(a) and (b).

Based on the actual lateral friction signal waves obtained from the two samples, we compare the simulation results and the actual friction signal; the results are summarized in Table 2.

**Table 2.** Relevant data of the simulation zigzag and armchair orientation parameters using in the simulation.

Lattice orientation type	Number of wave peaks in simulation result	Number of wave peaks in actual friction signal	Corresponding trajectory of actual friction signal	Shape of the wave in simulation result	Shape of the wave in actual friction signal
zz-1	10/7	10	A′-B′-C′-D′-E′	Saw-tooth wave	Saw-tooth wave
zz-2	13//11	11	F-G-H-I-J	Saw-tooth wave	Saw-tooth wave
ac-1	6/5	6	M′-N′-P′-Q′	Combination of two types of saw-tooth wave varying in height	Combination of two types of saw-tooth wave varying in height
ac-2	7	7	R-S-T ( R′-S′-T′)	Combination of two types of saw-tooth wave varying in height	Combination of two types of saw-tooth wave varying in height

According to Table 2, most of the data in the real signal profile exhibit consistent correlation with the simulation results with respect to the shape and the numbers of wave peaks. However, inconsistency between the simulation and the actual signal in the number of wave peaks is observed. The simulation is based on the assumption that each lattice direction may contain two trajectories. But in realistic friction experiments, only one of the postulated motion curves exists. According to the number of wave peaks in the actual friction signal, the possible paths for zz-1, zz-2, ac-1 and ac-2, are A′-B′-C′-D′-E′, F-G-H-I-J, M′-N′-P′-Q′, and R-S-T, respectively. The theoretical result is simulated under ideal conditions, whereas the experimental data with uncertainties leads to bias in various levels.

### 4.3. Power spectrum analysis of the friction signal variation in zigzag and armchair orientations

The direct comparison between the simulation result and the actual friction signal demonstrates the validity of the established motion model for the probe motion on the MoS_2_ surface. We conducted an extensive analysis of the actual friction signals between the two samples. As the AFM and LFM experiments are performed under ambient conditions, several external factors, such as alteration of the experimental parameters, environmental noise and condition of the apparatus, affect the experimental results to varying degrees. Hence, the actual friction signals include disturbances, and direct comparison is not suitable. To address this problem, an appropriate technique should be selected.

**Figure 9.**
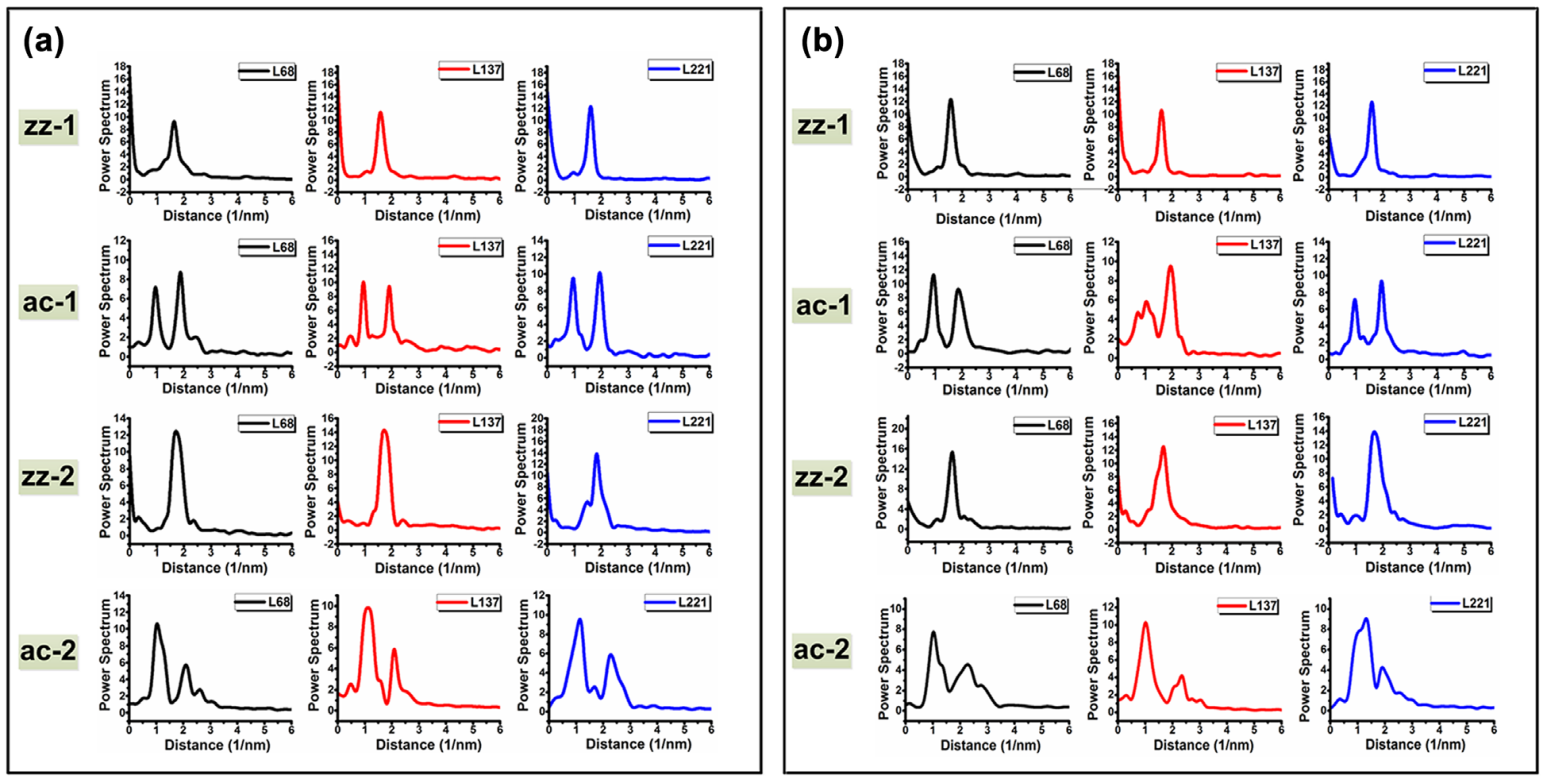
Power spectrum diagrams of the lateral fiction signal for (a) Sample-1 and (b) Sample-2. The waves in black, red and blue indicate the power spectra obtained from friction signal at the 68th, 137th and 221st rows of the atomic image, respectively.

FFT is a computationally effective technique in signal processing.[48] With the aid of FFT, the irrelevant external influences we mentioned above could be reduced and even eliminated. For these reasons, we employ this approach to process all of the waves in the various lattice orientations obtained from the friction atomic image. After processing the original friction signal waves in Figure 8(a) and (b), power spectra of each lattice orientation can be obtained, as shown in Figure 9(a) and (b).

Figures 9(a) and (b) show the distinct variation of the actual friction signals. For zz-1 and zz-2 lattice orientations, only one major peak appears in the corresponding power spectrum diagrams, whereas for ac-1 and ac-2 lattice orientations, the number of major peaks is generally two. This phenomenon is reasonable considering the shapes of the waves listed in Table 2. However, to thoroughly explain this phenomenon, we need to return to the modified interaction potential. In view of the physical structure of MoS_2_ and the relationship between ay and axis $a_{y}=\sqrt{3}a_{x}$, Equation 3 can be re-written as:

(5) $${\text{V(x}}_{t},y_{t})=V_{0}\cos\left(\frac{2\pi }{R^{*}}x_{t}\right)\cos\left(\frac{2\pi }{\sqrt{3}R^{*}}y_{t}\right)$$

In addition, *x*_*t*_ and *y*_*t*_ can be replaced by *x*_*t*_ = *r* cos *θ*,*y*_*t*_ = *r* sin *θ*. By substituting these two relations into Equation (5), the inherent frequency depending on the angle can be obtained:

(6) $$w_{1}=\left|\left(\cos\theta +\sin\theta /\sqrt{3}\right)/R^{*}\right|$$

(7) $$w_{2}=\left|\left(\cos\theta -\sin\theta /\sqrt{3}\right)/R^{*}\right|$$

By substituting the corresponding angle and the value of *R* ^*^, the frequency in various lattice orientations can be obtained. For zz-1 and zz-2, $w_{1}=w_{2}=\frac{1}{R^{*}}=\frac{1}{R\cos\theta }$. The angle of ac-2 is replaced by 30°, as discussed previously. Then, for ac-1 and ac-2, $w_{1}=\frac{2}{\sqrt{3}\text{R}}$, $w_{2}=\frac{1}{\sqrt{3}\text{R}}$. Only one frequency is obtained in zz-1 and zz-2, and two different frequencies are obtained in ac-1 and ac-2, which explains the one or two major peaks in the corresponding power spectrum diagrams.

### 4.4. Friction force in armchair and zigzag lattice orientations of two types of MoS_2_ multilayers

**Figure 10.**
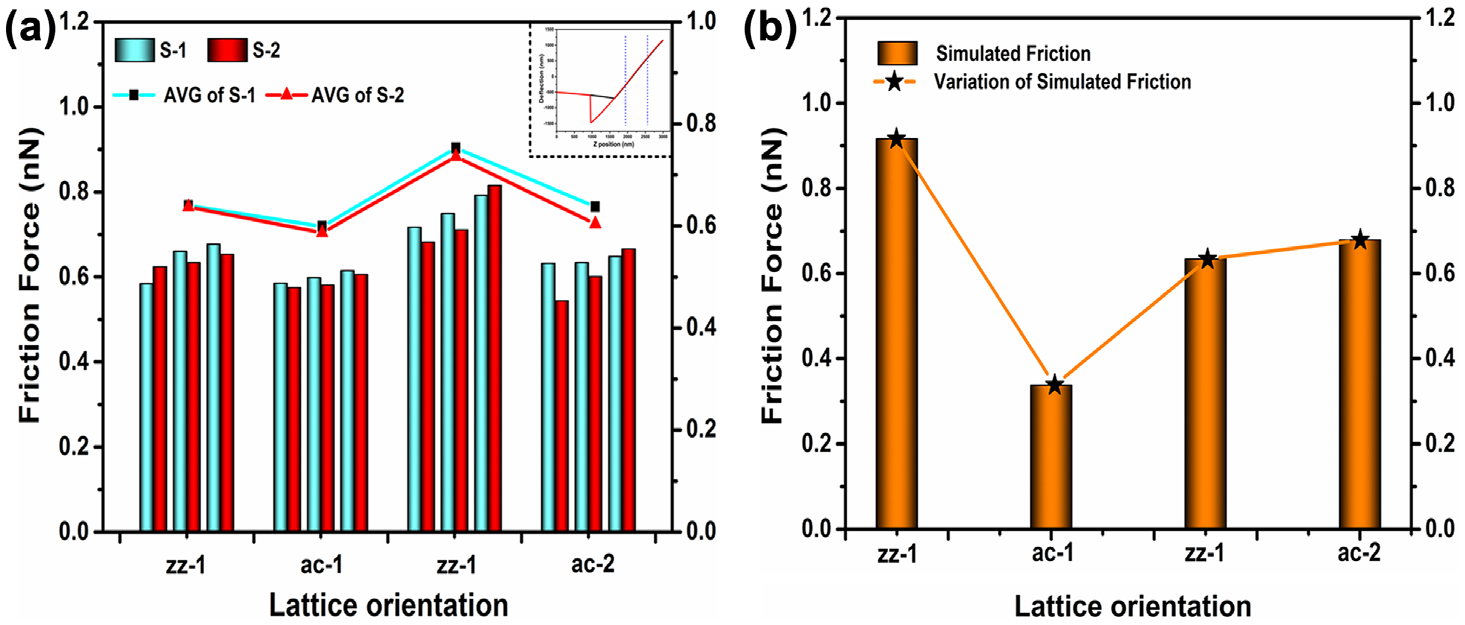
Friction force in multilayer MoS_2_ films. (a) Measured (inset: force curve of the probe) and (b) Simulated.

Finally, we discuss the atomic-scale friction force of multilayer MoS_2_ films. When the probe slides on the surface of MoS_2_ films, the lateral force *F*_1_ applied to the rectangular cantilever can be calculated by the following equation:[49]

(8) $$F_{l}=\frac{Gwt^{3}}{3l(h+t/2)}S_{l}V_{l}$$

where G is the shear modulus of the silicon nitride cantilever, and *w*, *l* and *t* are the effective width, length and thickness of the cantilever, respectively. *h* is the height of the probe. MLCT-B probes have the following dimensions and material constant: *w* = 2.1 × 10^–5^ m, *l* = 2.1 × 10^–4^ m, *t* = 0.55 × 10^–6^ m, *h* = 4.95 × 10^–6^ m, and *G* ≈ 0.53 × 10^11^ N m^–2^. *S*_*l*_ is the lateral sensitivity of the photodetector, and *V*_*l*_ is the corresponding photodetector lateral voltage output. According to the laser beam mechanism, the displacement of the laser spot on the photodetector is determined by the distance between the reflecting point and spot position on the photodetector and the reflecting cantilever’s angular deflection. The distance can usually be regarded as a constant; then, the lateral sensitivity *S*_*l*_ can be assumed to be equal to the normal sensitivity *S*_*n*_ based on the hypothesis that the photodetector is rotationally symmetric. Then, *S*_*l*_ can be calculated by:

(9) $$S_{l}=S_{n}=\frac{3}{2l}\frac{\text{d}x_{n}}{\text{d}V_{n}}$$

where $\frac{\text{d}x_{n}}{\text{d}V_{n}}$ can be derived from the slope of the force curve, which is presented in the inset of Figure 10(a). According to the calculation, $\frac{\text{d}x_{n}}{\text{d}V_{n}}=130.51\text{nm}V^{1}$. Then, the friction force of various lattice orientations can be calculated from the following equation:

(10) $$F_{l}\approx 0.052n\text{Nm}-1{\text{V}}^{-1}\times V_{l}$$

The actual friction forces of two MoS_2_ films in various lattice orientations are displayed in Figures 10(a) and (b). As shown in Figure 10(a), the average actual friction force in the thinner film, S-1, is higher than that of the thicker film, S-2, which is consistent with published reports.[50]

For more proof, we calculated the friction force of the simulation results. Based on the data displayed in Ffigure 8, the simulated friction force can be directly obtained by averaging the absolute values for various lattice orientations. The simulated friction force is displayed in Figure 10(b).

As shown in Figure 10(a) and (b), the friction force varies with the lattice orientations in MoS_2_. The discovery of this phenomenon will play a critical role in building a closed-loop fabrication strategy with real-time force sensor feedback to detect and control the lattice orientation. We also observed that the friction force in the actual situation does not perfectly match with the simulated results in terms of the friction force distribution in zigzag and armchair lattice orientations. The inconsistency is due to unavoidable experimental error. The exact reason remains unclear.

## 5. Conclusions

In summary, highly distinct, atomic-scale friction in zigzag and armchair lattice orientations of MoS_2_ is revealed through simulation and experiment. In particular, the friction signals of the two orientations are thoroughly compared. First, to reflect the relationship between the lateral friction and the lattice orientation, we propose the motion trajectories of the probe in both zigzag and armchair orientations and establish a comprehensive motion model. Then, the simulation of the friction force is completed using Matlab software. Furthermore, an AFM and LFM based experiment is conducted to obtain the actual friction signal. The consistency between the simulation results and the experimental data confirms the validity of the friction modeling for MoS_2_. By employing the FFT technique, the power spectra of the lateral friction in zigzag and armchair orientations are analyzed, which provides a more convenient and intuitive comparison. Finally, the actual and simulated friction forces are calculated. Although some discrepancies exist between the two results, the force variation dependence on the lattice orientation may hint a corresponding orientation detecting strategy in the future.

Our results may help to develop a reliable and effective method to experimentally differentiate these two important lattice orientations. The theoretical and experimental demonstration provides solid evidence of regular atomic-scale friction variation dependence on zigzag and armchair lattice orientations. Moreover, the distinct characteristics in both lattice orientations provide the possibility of establishing an effective lattice chirality detecting method or developing a real-time force sensing and feedback mechanism, which would be potentially useful for MoS_2_ and other two-dimensional materials.

## Notes on Contributors

*Meng Li* is currently working toward the Ph.D. degree from the State Key Laboratory of Robotics, Shenyang Institute of Automation, Chinese Academy of Sciences, Shenyang, China. Her current research interests include the atomic-scale research on the nano materials MoS_2_ and nano electrical devices.

*Jialin Shi* is currently working toward the Ph.D. degree from the State Key Laboratory of Robotics, Shenyang Institute of Automation, Chinese Academy of Sciences, Shenyang, China. His current research interests include ultrasonic vibration-assisted AFM technique and nanomanipulation based on nanorobots.

*Lianqing Liu* is currently a Professor at the Shenyang Institute of Automation. His current research interests include nanorobotics, intelligent control, and biosensors.

## Disclosure statement

No potential conflict of interest was reported by the authors.

## Funding

This work was supported by the National Natural Science Foundation of China [grant number 61522312], [grant number 61375107] and the CAS FEA International Partnership Program for Creative Research Teams.
